# Immunomodulation by Helminths: Intracellular Pathways and Extracellular Vesicles

**DOI:** 10.3389/fimmu.2018.02349

**Published:** 2018-10-12

**Authors:** Amin Zakeri, Eline P. Hansen, Sidsel D. Andersen, Andrew R. Williams, Peter Nejsum

**Affiliations:** ^1^Department of Clinical Medicine, Faculty of Health, Aarhus University, Aarhus, Denmark; ^2^Department of Veterinary and Animal Sciences, Faculty of Health and Medical Sciences, University of Copenhagen, Frederiksberg, Denmark

**Keywords:** extracellular vesicle, helminths, immunosuppression, intracellular pathways, pattern recognition receptors

## Abstract

Helminth parasites are masters at manipulating host immune responses, using an array of sophisticated mechanisms. One of the major mechanisms enabling helminths to establish chronic infections is the targeting of pattern recognition receptors (PRRs) including toll-like receptors, C-type lectin receptors, and the inflammasome. Given the critical role of these receptors and their intracellular pathways in regulating innate inflammatory responses, and also directing adaptive immunity toward Th1 and Th2 responses, recognition of the pathways triggered and/or modulated by helminths and their products will provide detailed insights about how helminths are able to establish an immunoregulatory environment. However, helminths also target PRRs-independent mechanisms (and most likely other yet unknown mechanisms and pathways) underpinning the battery of different molecules helminths produce. Herein, the current knowledge on intracellular pathways in antigen presenting cells activated by helminth-derived biomolecules is reviewed. Furthermore, we discuss the importance of helminth-derived vesicles as a less-appreciated components released during infection, their role in activating these host intracellular pathways, and their implication in the development of new therapeutic approaches for inflammatory diseases and the possibility of designing a new generation of vaccines.

## Introduction

### Host-parasite interactions (live infection, excretory secretory molecules, and extracellular vesicle)

Parasitic worms (helminths) constitute a very successful group of pathogens that have evolved a number of unique host adaptations (1). Despite their large size, and local or systemic migration throughout the host body, the worms only elicit limited inflammation in invaded tissues and install an immunoregulatory environment which ensures their survival (1). Such a masterful adaption is ascribed to their long coevolution with the hosts enabling them to perform an effective modulation of the immune system (2). Of note, this mutual relationship between worm and host evolves to reciprocal beneficial outcomes, as deworming can result in the emergence of immune-related disorders along with clinical manifestations (3). Various mechanisms have been identified by which helminths restrain host immune responses including expansion of regulatory cells (4), induction of apoptosis in immune cells (5), manipulation of pattern recognition receptors (PRRs) and downstream signaling (6), and suppression of Th1/Th2 cells and associated cytokines (7). However, there are likely to still be complex and unknown aspects of the strategies underpinning this sophisticated interface which are yet to be investigated (8).

In recent years, the regulatory functions of helminths and their potential ability to ameliorate inflammatory diseases have received much interest (8). Seminal research in this area mostly derives from the hypothesis of Strachan et al. who suggested an inverse relationship between sanitation and prevalence of allergy in different societies, the so-called “hygiene hypothesis” (9, 10). Based on this hypothesis, changes in or eradication of infections can lead to dysregulation of host immune responses, paving the way for allergic and autoimmune disorders (11).

A number of animal models along with some human pilot studies have evaluated the effects of live helminth infections on various inflammatory and autoimmune diseases, such as experimental autoimmune encephalomyelitis (12), asthma (13, 14), anaphylaxis (15) and inflammatory bowel disease (IBD) (16, 17). These experimental studies provided promising results concerning the beneficial effects of helminth infections on allergic and autoimmune diseases through stimulation of Treg cells, activation of toll-like receptor (TLRs), and induction of anti-inflammatory cytokines, such as TGFβ and IL-10 (18). Promising results obtained in both human and animal studies prompted clinical evaluations (16), but due to potential deleterious consequences and side effects which live worms may cause for humans, investigations have also focused on characterizing and recognition of helminth-derived products (HDPs) via exploiting high-throughput assays and omics-based techniques, such as proteomics (19, 20).

There are a number of studies indicating that many HDPs possess immunoregulatory properties. For example, the tapeworm, *Echinococcus granulosus* is able to bypass host immunosurveillance and polarize immune response toward the regulatory state (21). Antigen B (AgB) and sheep hydatid fluid (SHF) are two major components by which *E. granulosus* suppresses dendritic cell (DC) maturation and monocyte differentiation, resulting in reduced anti-parasite responses (21). Likewise, a well-known compound with remarkable regulatory functions is the phosphorylcholine-containing glycoprotein, ES-62 released by the filarial worm, *Acanthocheilonema viteae*, which has widely been investigated by Harnett and collogues (22). In addition, glycan-based compounds, such as Lacto-N-fucopentaose III /LewisX from helminths have been found to be central molecules eliciting Th2 responses and orchestration of immunoregulation through the involvement of C-type lectin receptors (23). Interestingly, some worms, such as the whipworm, *Trichuris suis* can forestall pro-inflammatory responses in human DCs (24). *T. suis* has been found to possess a high level of lipid-based biomolecules, such as prostaglandin (PGE2) which impairs TLR4-associated myeloid differentiation primary response protein 88 (MyD88) and the TIR-domain-containing adaptor-inducing interferon-β (TRIF) signaling (25, 26). Similarly, there is evidence showing that helminth defense molecules contribute to immunomodulatory outcomes of parasitic infections via targeting innate immunity (27). However, the study of HDPs is still a major research area and fractionating HDPs and subsequent detailed studies have opened a new avenue for ongoing investigations.

Recently, extracellular vesicles (EVs) have emerged as a previously unappreciated entity of HDPs which may play a crucial role in parasite immunomodulation. These “magic bullets” have encouraged investigators to unravel their role in pathogenicity, invasion, and longevity of parasitic infections (28). Currently, EVs have shown that may be central in the host-parasite interplay and intracellular communication (29). During infection, the immune system is constantly interacting with a wide range of helminth-derived products including EVs which eventually results in either immune stimulation or immunoregulation. For example, it has recently been documented that parasite EVs can manipulate macrophage activation and regulate inflammatory responses (30, 31). The intercellular delivery of EV-associated RNAs, such as microRNAs, has identified them as important means for inducing epigenetic modifications in intracellular signaling and post-transcriptional regulation of gene expression (30, 32).

In this review, we aim to elaborate modulation of intracellular pathways, mainly in antigen presenting cells (APCs), by which HDPs polarize and suppress host immunity. Moreover, we suggest that understanding the intracellular outcomes upon interaction with HDPs will provide a broad insight into the possible interactions between EVs (as an important component of HDPs) and host intracellular machinery. The putative pathways enabling EVs to impose immunomodulatory effects on host immunity are highlighted. Furthermore, the implication of these vehicles in the development of new therapeutic approaches against inflammatory responses and possibilities of designing a new generation of vaccines based on EVs are discussed.

## Helminth-derived products (HDPs) as potent immunomodulators

### How HDPs polarize immune responses by targeting intracellular pathways

Helminths have evolved sophisticated mechanisms to target intracellular machinery in host cells (33). They have shown a remarkable ability to induce a tolerogenic immune microenvironment by releasing an array of bioactive materials (33). A large body of literature has identified HDPs as powerful modulators of inflammatory signals comprising an impressive range of molecular pathways elicited against parasites (33). HDPs, in total and as individual compounds, play a central role establishing a beneficial niche for the parasite via an effective manipulation of the host immunity to engage a receptor, degrade intracellular molecules, and interfering with essential signals (34). However, the majority of intracellular pathways targeted by these biomolecules are poorly described, but in the following, we focus on innate receptors as important sensors which are targeted by HDPs.

Pattern recognition receptors (PRRs) are one of the most important immune receptors, and their signaling is now becoming more apparent in regulation of immune responses (35). PRRs are a family of highly sensitive extra and intracellular sensors including Toll-like receptors (TLRs), nucleotide-binding oligomerization domain (NODs)-like receptors (NLRs), retinoic acid-inducible gene-like receptors (RIG-like receptors), and C-type lectin receptors (CLRs) (35). They are widely expressed by immune cells, in particular, those responsible for immunosurveillance, such as DCs and macrophages. PRRs are able to trigger a complex of intracellular crosstalk resulting in DC maturation and T cell priming (36).

Since HDPs are mostly rich in glycan-based products, such as glycoprotein along with lipid structures, TLRs and CLRs have been found to be predominantly targeted by these antigens during immunomodulation and hyporesponsiveness (37). HDPs not only alter the expression of TLRs but also masterfully manipulate their intracellular signaling, reflecting a strict control over host immunity by helminths (6). Interfering with these intracellular pathways, which are main drivers for priming inflammatory responses, suggests that these extracellular parasites can release substances modulating early responding cells in innate immunity (38). Despite the general view that microbial components can engage PRRs and thereby activate DCs, it is well-known that DCs exposed concurrently to HDPs and TLR agonists, such as viral or bacterial products do often not express markers associated with classical maturation (39, 40). For instance, murine DCs treated with soluble SEA fail to express MHC-II, costimulatory molecules, and proinflammatory cytokines in response to LPS (40). In the same way, the release of antigen B (AgB) (a hydatid cyst-derived antigen) by *E. granulosus* prevents upregulation of LPS-induced CD80, CD86, and TNFα in DCs (21), monocytes, and macrophages via an IL-10 independent manner (41).

Intriguingly, HDPs can also modulate TLRs signaling to prime a tolerogenic phenotype of DCs that produce anti-inflammatory cytokines (42). *Schistosoma mansoni* and released eggs can produce bioactive antigens, such as lysophosphatidylserine, lacto-N-fucopentaose III (LNFPIII), and double-stranded RNA (dsRNA) which attenuate inflammatory responses by targeting TLRs. LNFPIII has also been reported to induce DCs maturation and Th2 response polarization through CLRs (23). Another biomolecule that interacts with TLRs is ES-62 released by *A. viteae* which targets TLR4 on host immune cells. ES-62 is able to interfere with the downstream signalings mediated by TLR4, and through which diminishes the production of inflammatory mediators (22).

**Figure 1:**
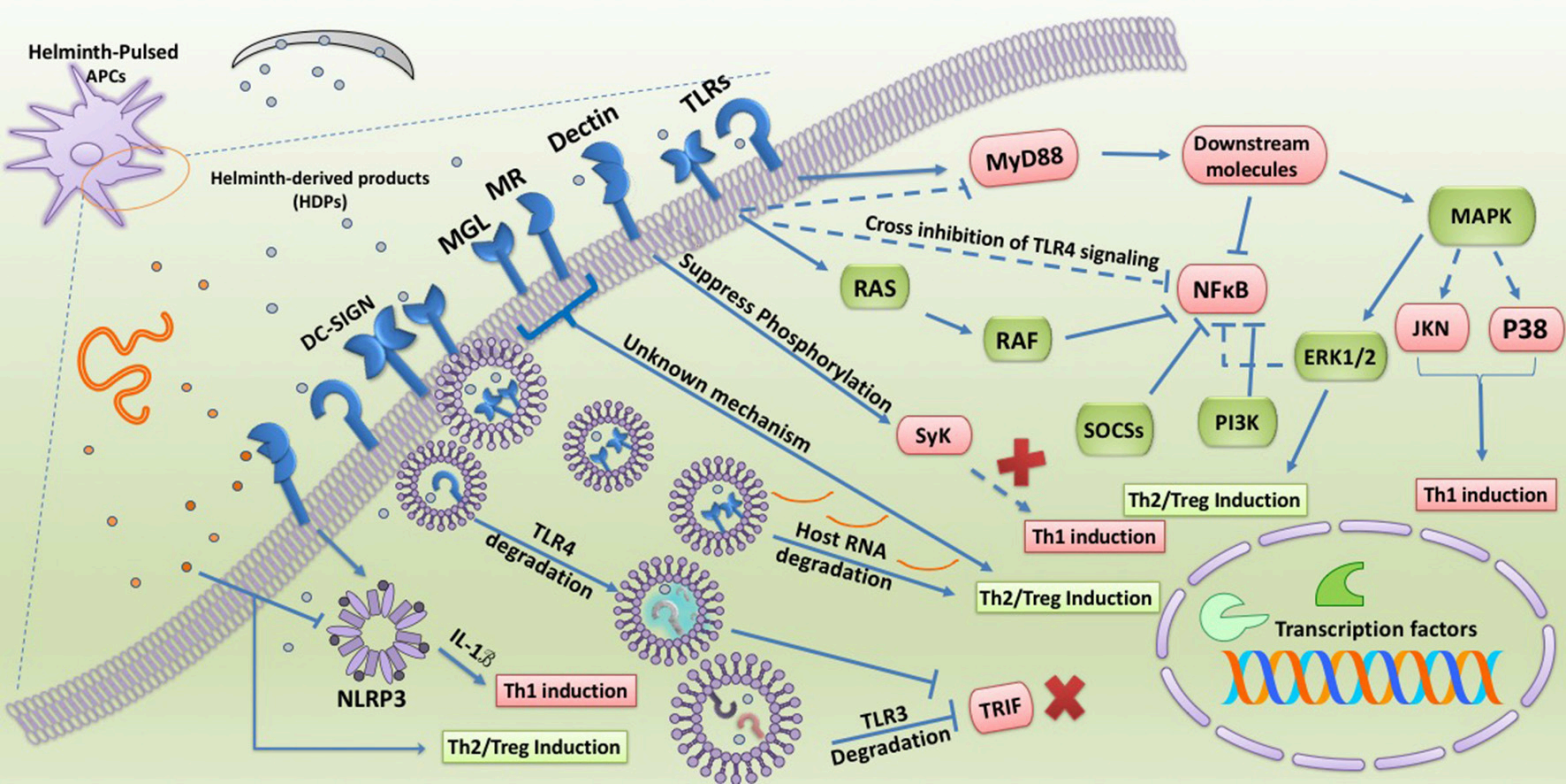
Involvement of TLRs and CLRs during interaction with HDPs. TLRs and CLRs are widely targeted by HDPs during induction of immunomodulation and hyporesponsiveness in APCs. HDPs not only alter the expression of TLRs and CLRs in APCs but also masterfully manipulate their intracellular signaling. Some HDPs are able to redirect TLR4 signaling toward MAPK pathway and ERK1/2 activation supporting Treg/Th2 induction. In addition, co-engagement of DC-SIGN along with TLR4 enables HDPs to trigger unknown intracellular pathways which cross-inhibit MyD88 and NFκB activation. HDPs can further restrain NFκB activity via DC-SIGN-mediated RAF signaling along with upregulation of negative regulators of TLRs signaling, such as SOCSs and PI3K. Obviously, strict inhibition of NFκB as the main transcription factor supporting inflammation results in prevention of priming Th1 cells. Other CLRs have been reported to participate in priming Treg/Th2 cells upon stimulation by HDPs. For example, some HDPs suppress phosphorylation of Dectin1/2-induced Syk molecule and through which inhibit deviation of immune response toward Th1. On the other hand, MR and MGL upon activation by HDPs through an unknown mechanism support Treg/Th2 differentiation. Degrading host key intracellular molecules is another strategy that HDPs exploit to reprogram host immunity. Omega-1, ES-62, and FheCL1 by degrading host mRNA, endosomal TLR4, and TLR3, respectively, not only strengthen Treg/Th2 responses but also forestall anti-parasite immunity. NLRP3 also has been revealed to be targeted by some HDPs to modulate inflammatory responses. However, there has been reported that some HDPs are able to fight anti-worm immunity by stimulation of NLRP3 leading to release of IL-1B and Th1 amplification.

Likewise, many other HDPs, such as body fluid from adult *Ascaris suum*, have also been documented to induce hyporesponsiveness in human APCs treated with LPS and modulate different human macrophage phenotypes (43, 44). Although HDPs mainly seem to impair TLR4-associated inflammatory responses, it appears that these components tend to target manifold pathways in TLRs and CLRs signaling. Recent bioinformatics-based data has suggested that HDPs constitute a myriad of molecules with complex structures (20). Thus, recognition of a target on DCs would be essential to dissect and identify the major immunosuppressive functions. Here we focus on the main PRR-associated intracellular machinery that is altered by HDPs to favor helminth survival and persistence in the host (Figure 1).

#### TLR signaling (map kinases and NF-κ B cascade)

Mitogen-activated protein kinases (MAPKs) are a group of highly important molecules orchestrating the production of different cytokines via involving various downstream accessory proteins in DCs (45). The MAPK pathway is one of the main signaling cascades induced as a result of TLRs stimulation (45). Different kinases contribute to MAPK signaling including the extracellular signal-related kinases 1 and 2 (ERK 1/2), c-jun NH2-terminal kinase (JNK), and p38 MAPK. Activation of these molecules results in DC maturation, cytokine production, and gene expression via stimulation of transcription factors, such as activating protein 1 (AP-1), nuclear factor-κB (NF-κB), and IFN regulatory factors (IRFs) (45). ERK1/2 signaling mostly mediates Th2 response and DCs hyporesponsiveness due to stabilization of the c-fos transcription factor which suppresses IL-12 production (46, 47), whereas JNK and p38 are mostly associated with Th1 responses and DCs activation (47). In support of this, blocking ERK1/2 pathway by the specific inhibitor U0126 up-regulates IL-12 and suppresses TLR2-induced IL-10 production (47). Furthermore, it has been reported that ERK1 knockout mice spontaneously develop autoimmunity (48). On the other hand, two important adaptor molecules associated with TLRs signaling are MyD88 and TRIF, and TLR-induced activation of these molecules is responsible for the expression of genes encoding inflammatory mediators, such as IL-12 and TNFα (36). A number of helminths release molecules that are capable of triggering anti-inflammatory responses via interference with these downstream pathways (38, 49, 50). For example, soluble products of *T. suis* not only suppress transcription of essential molecules orchestrating both MyD88 and TRIF pathways in LPS-treated DCs, but also restrain TLR4 expression preventing DC maturation (26). The precise mechanisms by which HDPs influence these intracellular events are currently the subject of intense investigation. However, interesting data is becoming available regarding inhibitory effects of some HDPs, such as ES-62 and LNFPIII on key molecules involved in these inflammatory pathways.

One of the most important molecules that is targeted by HDPs to subvert TLRs signaling is ERK1/2. This intracellular component plays an essential role in mediation of anti-inflammatory functions of ES-62 and LNFPIII. In fact, both LNFP-III and ES-62, through engagement of TLR4 on DCs, induce Th2 responses (38, 49, 50). Although the capacity of TLR4 in skewing immune response toward Th2 seems surprising, various mechanisms might be at play and might explain the strikingly different response to LPS and some HDPs (51). The main explanations for this phenomenon are selective stimulation of ERK1/2 signaling and involvement of different co-receptors by HDPs which eventually interfere with TLR4-induced inflammatory signals in DCs (38, 51). In addition, it should be noted that TLR4 signaling can be conducted by two distinct of adaptor molecule pathways (TRIF and MyD88), however, it is still obscure which adaptor molecule is responsible for induction of immunoregulatory signals and how it is selectively activated by HDPs (38).

There is strong evidence that activation of ERK1/2 signaling inhibits a Th1 response and instead polarizes immune responses toward anti-inflammatory and Th2 response both *in vivo* and *in vitro* (45, 52). LNF-PIII is one the most powerful HDPs in supporting ERK signaling which significantly induces phosphorylation of ERK and TLR4-dependent differentiation of naive DCs to a DC2 phenotype (50). It seems that such a dysregulation of TLR signaling with a bias toward ERK1/2 is an effective strategy employed by the parasite to suppress Th1 responses and polarization of host immunity. In addition, *Trichinella spiralis* muscle larvae secrete bioactive components which strongly induce transient ERK1/2 signaling, thereby priming Th2/Treg-inducing DCs (53). However, different stages of this nematode have also been shown to arrest NF-κB translocation and the phosphorylation of p38 and ERK1/2 in LPS-treated J774A.1 murine macrophage cell line (54).

To further explore the mechanism of these biomolecules on immune cells, it is necessary to compare the signaling pathway(s) triggered by these antigens and LPS (38). First, stimulation of TLR4 by LPS results in triggering three different aforementioned MAPK cascades (ERK1/2, JNK, and p38 MAP kinases) along with NF-κB pathway activation which ultimately leads to the release of pro-inflammatory mediators (55). In contrast to the response to LPS, it has been shown that LNFPIII and ES-62 are able to induce mainly ERK1/2 signaling without significant stimulation of p38 and JNK. Second, further molecular dissection revealed that these HDPs not only slightly activate NF-κB signaling relative to LPS, but also shorten NF-κB longevity by stabilizing its inhibitor (50, 56). Third, the suppressive functions of ES-62 have been ascribed to inhibition of p38 and JNK, which play a central role in the production of Th1-associated cytokines, such as IL-12, IL-6, and TNFα (56, 57). Fourth, it should be noted that another explanation for such a difference in signal transduction between LPS and ES-62 might be due to the discrepancy in ubiquitylation of TLR4 and/or downstream signaling complexes (57). These data support the notion that some HDPs can elicit Th2 response by TLR4 stimulation in a manner distinct from customary agonists like LPS, which is a strong Th1 inducer.

The modulatory effects of ES-62 are not restricted only to the skewing of TLR4 signaling toward ERK1/2. Melendez et al. showed that ES-62 can also degrade key molecules involved in FcεRI signaling on mast cells via TLR4 engagement (58). Multiple downstream molecules are activated upon binding IgE to FcεRI, such as protein kinase Cα (PKCα), phospholipase D-coupled, and sphingosine kinase 1 (SPHK1) which subsequently trigger NF-κB signaling. In comparison to LPS, which strengthens (FcεRI)-mediated signaling, ES-62 directs PKCα toward degradation via a TLR4-dependent and proteasome-independent manner, suppressing mast cell activation in response to IgE stimulation (58). Importantly, other members of the PKC family (PKCβ, PKCδ, PKCι, and PKCζ) can also be degraded by ES-62 (58, 59). Among them, PKCδ is regarded as a TLR4-mediated pathway molecule, which plays an important role in full activation of LPS-induced inflammation (60). Eason et al. have recently reported that despite LPS, ES-62 can exert downregulation and autophagolysosomal degradation of PKCδ in DCs to undergo autophagy (60).

Finally, a significant discrepancy between LPS and ES-62 stimulation of TLR4 relates to intracellular trafficking of this receptor. It has been shown that ES-62 can trigger TLR4 trafficking to a distinct caveolae lipid raft route leading to TLR4 degradation, whereas the route through which LPS induces TLR4 trafficking is associated with the promotion of Th1 and inflammatory responses (58). Owing to promising results achieved from ES-62, analog molecules of ES-62 termed 11a, 11e, 11i, and 12b have been synthesized by Harnett and colleagues. These synthetic structures could experimentally suppress collagen-induced arthritis (CIA) by downregulation of key adaptor molecules in TLRs signaling, such as MyD88 and NF-κB (61–63).

Of note, other helminthes, such as *S. mansoni* and *Ascaris lumbricoides* can induce an imbalance in the intracellular signaling of LPS-treated human DCs via engagement of TLR2 and high stimulation of the ERK pathway. Given the fact that ERK1/2 signaling can increase c-Fos stabilization and IL-12 inhibition (47), it has been demonstrated that *S. mansoni* and *A. lumbricoides*-derived phospholipids support Th2 polarization via induction of imbalance in TLR2 signaling by strengthening ERK pathway in human monocyte-derived DCs (64). Interestingly, soluble SEA through TLR2 signaling induces a crosslink manipulation on the expression of co-inhibitory-associated genes, such as programmed death-ligand 1 (PD-1) and PD-L2 on murine BMDCs and CD4^+^ T cells, respectively. Upregulation of PD-1 and PD-L2 expression by egg antigens is a TLR2-dependent mechanism leading to anergy and hyporesponsiveness during interaction between macrophages and CD4^+^ T cells (65).

In addition, Correale et al. have suggested that SEA can activate several genes associated with retinoic acid synthesis, such as SOCS3 and IL-10R in DCs, via engagement of TLR2 and activation of ERK1/2 signaling (66). Importantly, egg antigen-treated DCs have been shown to strongly prime Tregs and suppress production of inflammatory cytokines, indicating a clear polarization by targeting TLR signaling. Mechanistically, Agrawal et al. suggested that immunoregulatory function of egg antigen is mediated via ERK1/2-induced c-Fos phosphorylation, as in the absence of c-Fos immune response were redirected toward Th1 (47).

Moreover, some HDPs, such as egg antigens condition DCs to prime Foxp3 Tregs via TLR2-dependent ERK1/2 signaling. This effect can also be observed with the antigen SJMHE1 from *S. japonicum* which increases proliferation and suppressive functions of Tregs by activating TLR2 (67–69). However, it is unknown how HDPs modify TLRs on Tregs to optimize their lifespan and suppressive activity, so further research is required to document the underlying molecular mechanism. Generally, TLRs signaling is mediated through two distinct pathways known as MAPK and NF-κB cascade. NF-κB signaling also plays an essential role in priming Th2 response, since the lack of NF-κB in DCs has been shown to result in Th2 impairment in the presence of egg antigens and LNFPIII (70, 71).

#### SOCSs, JAK/STAT, and PI3K

There is evidence suggesting that some HDPs can directly manipulate molecules controlling TLRs signaling, such as suppressor of cytokine signaling 3 (SOCS3) (66, 72). In this regard, it has been shown that *F. hepatica* produces biomolecules, such as tegumental coat antigens (FhTeg) and cathepsin L1 cysteine protease (FheCL1) which are capable of interfering with both the NF-κB and ERK/MAPK pathways in a manner distinct from other HDPs (72, 73). For instance, FhTeg can diminish the expression of inflammatory mediators in LPS-treated DCs via upregulation of a negative regulator of the TLRs pathway known as SOCS3 (72). On the other hand, FheCL1 is involved in neutralizing TLR3 signaling, as a TLR utilizing TRIF adaptor as an activator of downstream molecules. In fact, Donnelly et al. indicated that TRIF-dependent MyD88-independent signaling pathway is impaired by FheCL1 via degradation of TLR3 (73). Similar to FheCL1, SmCB-1 from *S. mansoni* has been found to enter the endosome and degrade TLR3. The protective effects of these antigens (FheCL1 and SmCB-1) against LPS-induced lethality were found to be mediated through MyD88-independent and TRIF-dependent pathways shared by both TLR4 and TLR3, suggesting the potential of HDPs as druggable targets due to their ability to disrupt intracellular pathways activated during inflammation (73). The TRIF-dependent pathway of TLR4 signaling can also be inhibited by *T. spiralis*-derived antigens as a mechanism to orchestrate regulatory responses (74). *Brugia malayi* has also been shown to produce a component known as abundant larval transcript (ALT-2) which promotes type 2 immune response and attenuates IFN-dependent signals via activation of GATA-3 and SOCS-1 in macrophages (75). SOCS-1 has also been reported to be upregulated by SEA in human DCs exposed to LPS which was associated with inhibition of pro-inflammatory cytokines (76).

Apart from ES-62, another well-known and highly bioactive component from *A. viteae* with significant intracellular activity is AvCystatin (77). Generally, cystatins are regarded as protease inhibitors which have been detected in excretory-secretory products of most filarial nematodes (78). Macrophages and IL-10 have been found to be central in the immunoregulatory effects of AvCystatin on experimental models of colitis and asthma (77, 79). AvCystatin is able to induce regulatory macrophages (Mreg) representing remarkable suppressive effects on DCs via IL-10 dependent and cell-contact independent mechanism (79). Klotz et al. identified the molecular mechanism by which AvCystatin can reprogram the macrophage phenotype and suppresses inflammation. They suggest that AvCystatin is taken up by macrophages and through stimulation of dual specificity phosphatases (DUSPs), which are negative regulators of MAPK signaling and IL-10 expression, targets ERK1/2 and p38 to modulate downstream signals inducing regulatory responses (77). Through this mechanism, the immunoregulatory effects of AvCystatin are mediated via the phosphorylation of the CREB and STAT3 (77). In addition, some HDPs, such as dsRNA derived from *S. mansoni* egg can also modulate STAT1 signaling (80). This dsRNA is able to engage TLR3 and phosphorylate STAT1 in DCs supporting signaling pathway associated with type I IFN expression (80). Yang et al. have recently provided evidence suggesting that the immunomodulatory activity of *S. mansoni* egg antigens (SEA) and *S. japonicum* worm antigen (SWA) in myeloid-derived suppressor cells (MDSCs) is mediated through the Janus kinase/signal transducers and activators of transcription 3 (JAK/STAT3) pathway (81). They corroborated their findings by application of a JAK inhibitor (JSI-124) which abrogated immunomodulatory functions of SEA and SWA (81). Besides STAT1 and 3, STAT6 signaling has also been found to be manipulated by some HDPs, such as *T. spiralis*-derived cathepsin B-like protein (82). Liu et al. indicated that the recombinant form of this antigen (rTsCPB) can significantly restrain intestinal ischemia/reperfusion injury in mice by active reprogramming macrophages from an inflammatory (M1) to an alternative phenotype (M2) (82). One of the main intracellular signalings for such a phenotypic alteration is activation of STAT6 in M1 macrophages (82). Mechanistically, rTsCPB was shown to support upregulation of M2-associated markers and subsequently transition of M1 to M2 macrophage via STAT6-dependent manner, as inhibition of STAT6 restored disease severity and M1 domination (82).

Some parasites tend to target another pathway in TLR signaling known as phosphoinositide 3-kinase (PI3K) which is a strong inhibitor of TLR-induced IL-12 production and DCs maturation (77, 83, 84). Accordingly, this pathway has been revealed to shape immune response toward Th2 and IL-10 production. For instance, some protozoan parasites, such as *Giardia lamblia* and *Leshmania major*, along with *A. vitae*, are able to interfere with TLR-mediated DCmaturation via stimulation of PI3K signaling maturation (77, 83, 84). Importantly, both CLRs and TLRs can share ERK and PI3K signaling to redirect immune response toward Th2 during infection with helminths (85).

These data show how HDPs may impact TLR signaling, thereby bypassing inflammatory responses. The predominant pathway triggered by HDPs is ERK1/2 signaling which supports phosphorylation of the transcription factor c-Fos inducing modified Th2 and/or anti-inflammatory responses (46, 50, 56). Generally, it seems that one of the main mechanisms by which helminths minimize host tissue injury is suppression of innate immunity via modification of TLR signaling (6, 85).

#### C-type lectins receptor (CLRs) signaling

There is evidence suggesting the commencement of Th2 response can be initiated independent of the two adaptor proteins MyD88 and TRIF (86). In this case, it is expected that other PRRs beside TLRs also can initiate the Th2 response (37). As mentioned above, APCs express an array of receptors known as CLRs specialized in the recognition of glycans (87). CLRs can mediate different immunological processes including antigen uptake, intracellular trafficking, and priming innate immunity (87). Various types of CLRs are expressed by APCs including DC-specific ICAM-3 grabbing nonintegrin (DC-SIGN), macrophage galactose binding lectin receptor (MGL), mannose receptor (MR), and Dectin-1. DC-SIGN is involved in recognition of high mannose glycans and is able to stimulate TLR signaling (87). MGL and MR detect pathogens-associated mannose-containing glycans with high sensitivity (87). Recently, surfactant protein (SP)-D, collectin related to the family of C-type lectins, has also been reported to interact with carbohydrate-based compartments of worms, such as *Nippostrongylus brasiliensis* in the lung (88). Thawer et al. demonstrated that SP-D KO mice are unable to control *N. brasiliensis* in the lung, as this protein plays a key role in stimulation of Th2 responses and alternative activation of alveolar macrophages (alvM) which is essential for binding to and killing L4 parasites (88).

Importantly, CLRs play an important role in host-parasite interaction through recognition of glycan-based components in helminth-derived antigens (89). These parasite-derived glycans constitute versatile glycoconjugates with highly various structures targeting CLRs, and in turn skew adaptive immunity (90). For instance, N-linked glycoconjugates from *A. suum* extract engage DC-SIGN and MR to suppress LPS-induced DCs maturation and triggering inflammatory signals (89). Similarly, it has been shown that DC-SIGN, MR, and MGL can be involved in capturing and internalization of SEA (91–93). Also, some HDPs are quite similar to host glycans which, during interplay with CLRs of DCs, induce both Th2 suppression and Treg proliferation, establishing a tolerogenic state in host immune function (94). In this way, this mechanism can create a balance between Th1 and Th2 responses through the involvement of CLRs on DCs (37, 95). Unfortunately, limited data are available on the parasite components inducing immunological bias via the involvement of CLRs.

One of the main pathways shared by most CLRs to trigger downstream signaling is the spleen tyrosine kinase (Syk) pathway, which has been shown to contribute to DC priming and elicitation of inflammatory responses (96). Interestingly, *Heligmosomoides polygyrus* and its products elicit a strong downregulation of different CLRs including CLEC7A, 9A, 12A, and 4N along with suppression of Syk phosphorylation (96). In fact, it is suggested that the inhibitory effects of this worm on CLRs and Syk expression leading to induction of regulatory DCs in intestine and mitigation of colitis in infected mice (96). In another way, excretory-secretory products of *Taenia crassiceps* (TcES) have been found to inhibit DCs maturation in response to TLR4 and TLR9 agonist (97). Molecular dissections revealed that TcES are able to interfere with TLR signaling and induce tolerogenic DCs. Terrazas et al. delineated that TcES support Th2 response by activation of MGL, MR, and TLR2 (97). The reported that the major intracellular mechanism by which TcES prevent TLR4-mediate DCs maturation is by targeting c-RAF, which is a MAP3K acting on downstream pathways of the Ras family (97). Indeed, TcES significantly suppress downstream molecules, such as p38 and NF-κB by phosphorylation of c-RAF and consequently polarize immune responses toward a Th2 phenotype (97). It has also recently been observed that *T. crassiceps*-induced Ly6C^hi^ monocyte-derived alternatively activated macrophages (AAMs) express a high level of MR (CD206), PD-L2, and CCR2/CX3CR1 enabling them to prime Tregs and suppress experimental autoimmune encephalomyelitis in mice (98).

CLRs have also been suggested to associate with induction of Th2 responses by *Toxocara*-derived antigens (99). Among them, DC-SIGN is one of the most important receptors in recognition of *T. canis, F. hepatica*, and *B. malayi*-derived glycan products shaping the immune response toward a Th2/regulatory state (100–102). Rodríguez and colleagues have recently found that DC-SIGN plays a central role in priming Treg upon interaction with *F. hepatica*-derived glycans (102). The presence of mannose and fucose residues in *F. hepatica*-glycoconjugates can facilitate DC-SIGN stimulation and trigger intracellular pathway enabling DCs to prime Treg and inhibit proliferation of allogeneic T cells (102). Surprisingly, stimulation of DC-SIGN with *F. hepatica*-glycoconjugates activates a pathway in DCs which, during intracellular crosstalk with TLR-mediated signaling, induces IL-10 and IL-27 secretion in support of Treg expansion (102). A similar mechanism has been reported for immunoregulatory effects of *F. hepatica*-derived glycans upon engagement of MGL on both human monocyte-derived DCs (mo-DCs) and mMgl2^+^ CD11^+^ cells in mice (103). In this study, the authors suggest that a cross regulation between pathways due to co-stimulation of MGL and TLR4 with *F. hepatica*-derived glycans and LPS, respectively, which enables DCs to produce immunoregulatory cytokines and support Th2/Treg proliferation (103). MR can recognize *F. hepatica* tegumental antigens (FhTeg) and condition DCs to induce anergy in CD4^+^ T cells (104). In addition, this receptor is able to suppress DCs maturation or Th2 induction in response to *F. hepatica* total extract (105, 106). However, it is believed that MR is not solely involved in conduction of immunosuppressive function of FhTeg and other CLRs most likely also contribute to these interactions (107). In support of this, *F. hepatica* excretory-secretory products (FhESP) have been demonstrated to suppress T cell activation via Dectin-1 dependent upregulation of PD-L2 and IL-10 in macrophages (108). Also, the strong suppressive activity of FhESP has been ascribed to co-activation of MR and Dectine-1 in macrophages underpinned by high levels of TGF-β and IL-10 production (109).

SEA possesses an array of complex glycan-based products which are predominantly recognized and taken up by surface CLRs on DC including MR, MGL, DC-SIGN, and Dectin1, triggering SYK-mediated intracellular pathways (91–93, 110). Endocytosis of egg-derived antigens results in interference with TLR signaling in DCs (93). The most well-known antigens of *schistosoma* egg which stimulate CLRs are LEX-containing glycans, omega-1, lacto-N-fucopentaose III and lacto-N-neotetraose. LEX-containing glycans involve DC-SIGN on DCs and trigger intracellular signaling, thereby promoting Th2 responses (111). Moreover, plant-based reconstruction of omega-1 has recently been reported to provoke Th2 responses by engaging DC-SIGN (51). Omega-1 is a Lex-containing glycoprotein structure representing T2 ribonuclease (RNase) activity. The MR has also been displayed to capture and internalize omega-1 leading to Th2 polarization along with interfering protein synthesis via degrading host cell RNAs (112, 113). LNFPIII and lacto-N-neotetraose are potent suppressors of T cell proliferation and DCs activation through stimulation of IL-10 production (114). Interestingly, it has been indicated that the immunoregulatory functions of these glycan-based biomolecules are mediated via the MR and DC-SIGN (38, 49, 115). Of interest is a recent study by Kooij et al. which highlighted a critical role of MR in polarization of human classical monocytes toward regulatory phenotype (expressing SOCS1, IL-10, and TGFβ) upon interaction with soluble products of *T. suis* (TsSP) (116). In this study, MR was found to respond to TsSP and triggers a PKC-mediated signaling (mainly PKCδ) responsible for induction of anti-inflammatory monocytes (116). Also, glycan compounds in *T. spiralis* muscle larvae excretory-secretory antigens (ES L1) have been reported to be able to polarize host immunity toward Th2/Treg via corroboration of ERK1/2 signaling in DCs and production of IL-10 and TGFβ (117). However, the major receptor responsible for such modulation is obscure and possible candidates need to be investigated (117).

These data suggest that CLRs can strongly react to HDPs and condition DCs to redirect immune response toward Th2 immunity and/or an anti-inflammatory response (118). However, the precise shared pathway by TLRs and CLRs upon simultaneous activation leading to release of regulatory cytokines remains enigmatic. In the following, we discuss this topic, to which less attention has been given.

#### Co-involvement of TLRs and CLRs by HDPs

Interestingly, recent data suggest that the immunomodulatory functions of some HDPs are mediated via simultaneous involvement of TLRs and CLRs. In fact, signaling pathways of CLRs have shown to co-operate with TLRs which strengthen effective immunomodulation. For example, *S. mansoni* secretes glycolipids which co-involve DC-SIGN and TLR4 in human DCs (119). Regarding intracellular crosstalk, Meyer-Wentrup et al. suggested that TLR8-mediated production of inflammatory cytokines, such as IL-12 and TNFα can be forestalled by dendritic cell immunoreceptor (DCIR) as a C-type lectin receptor (120).

This complex mechanism suggests an intricate intracellular crosstalk between two distinct receptors which results in hyporesponsiveness and immunosuppression. Activation of CLR signaling can modify inflammatory responses via manipulation of TLRs signaling (121, 122). For instance, SEA and LNFPIII are among the well-known schistosome-associated antigens which have been reported to co-involve TLRs and CLRs in host DCs. Egg antigens can regulate TLR2, TLR3, and TLR4 signaling through stimulation of β-galactoside-binding lectin galectin 3 (40, 80, 123–125), while LNFPIII via interaction with DC-SIGN and MGL1(23, 126), cross-inhibits the TLR4 pathway (101). DC-SIGN also strongly directs the immune response toward Th2, Treg, and modulation of inflammatory reactions. In this regard, Geijtenbeek et al. showed that mannosylated components of Mycobacterial cell walls diminish TLR4-associated inflammation via DC-SIGN (122). Similarly, other pathogens seem to modify TLR-mediated inflammatory consequences through stimulating DC-SIGN-associated Raf-1 signaling (127).

Likewise, other glycan-based compounds released by *S. mansoni* are expected to modify inflammatory responses via co-involvement (128). The immunoregulatory activity of schistosome-derived lysoPS may be similar to zymosan, which co-engages TLR2 and Dectin-1 (a type of CLRs) on DCs and induces Treg priming along with IL-10 production (129). Stimulation of Dectin-2 by whole extracts of *S. mansoni* eggs was reported to trigger Syk and inflammasome signaling and thereby enable inhibition of TLR signaling (110). Apart from intracellular crosstalk, physical interaction between CLRs and other surface receptors is believed to affect their intracellular signaling leading to a significant modification in immune response (128). Generally, it seems that glycan-based materials released by helminths can modulate host immunity through manifold signaling stemming from different receptors, such as TLRs, CLRs, and likely other PRRs provoking both Th2 and regulatory responses (23, 130). Tracking the mechanisms through which HDPs induce Th2 and/or regulatory responses will provide new insight into recognition of molecules and targets that selectively stimulate ERK signaling, which may be beneficial in development of new generations of vaccines and therapeutics against inflammatory diseases.

#### Inflammasome

Inflammasomes constitute an intracellular platform possessing NOD-like receptor (NLR) family proteins which are highly sensitive to various PAMPs and DAMPs (131). These cytosolic sensors include different family of NLRPs and absent in melanoma 2 (AIM2) which play a key role in triggering inflammatory signals in most immune cells, but mainly in APCs (131). Stimulation of inflammasomes results in activation of a cascade signaling supporting active release of IL-1β and IL-18 (131). However, limited data is available on the interaction of helminths with inflammasome, but some studies have provided interesting results in this regard. For example, Rzepecka et al. reported that a synthetic analog of ES-62 known as SMA-12b is able to suppress arthritis in mice by prevention of inflammasome activation (132). Further in-depth molecular dissection on murine macrophages revealed the underlying mechanisms are mediated through downregulation of IL-1β and inflammasome-associated signals which strongly counters inflammasome-induced responses supporting arthritis (132). Some helminth infections and their products are able to activate inflammasome in order to restrict production of early released innate cytokines, such as IL-25 and IL-33 (133). For example, *H. polygyrus* has been found to instigate NLRP3 in intestinal lamina propria cells inducing IL-1β secretion and by this mechanism prevents Th2 response initiation, ILC2 expansion, and eventually helminth expulsion (133). The same scenario has recently been reported for *T. muris* as Alhallaf et al. unraveled a novel mechanism by which *T. muris* exosomes and ESP suppress anti-parasite immunity by targeting NLRP3 (134). This study showed that this worm and its released compounds actively counter Th2 response and worm expulsion by increasing NLRP3-mediated IL-18 both *in vivo* and *in vitro* (134). Thus, this intracellular sensor may be targeted by helminths to suppress Th2 responses via IL-1β and IL-18 (133, 134).

Likewise, a well-known component from *S. mansoni* called omega-1 has also shown to be able to activate NLRP3 via co-involvement of Dectin-1 in macrophages (135). However, it is unknown how modulating the inflammasome can facilitate infection. In the same line, Ritter et al. studied the inflammasome-associated activity of SEA (110). They suggested that egg antigens can simultaneously suppress TLR signaling and activate NLRP3. Mechanistically, egg antigens were found to require co-activation of Dectin-1/FcγR and downstream signaling (Syk kinase signaling) to irritate NLRP3 via activation of reactive oxygen species and potassium efflux leading to IL-1β secretion in BMDCs (100).

#### Targeting non-PRR signaling

**Figure 2:**
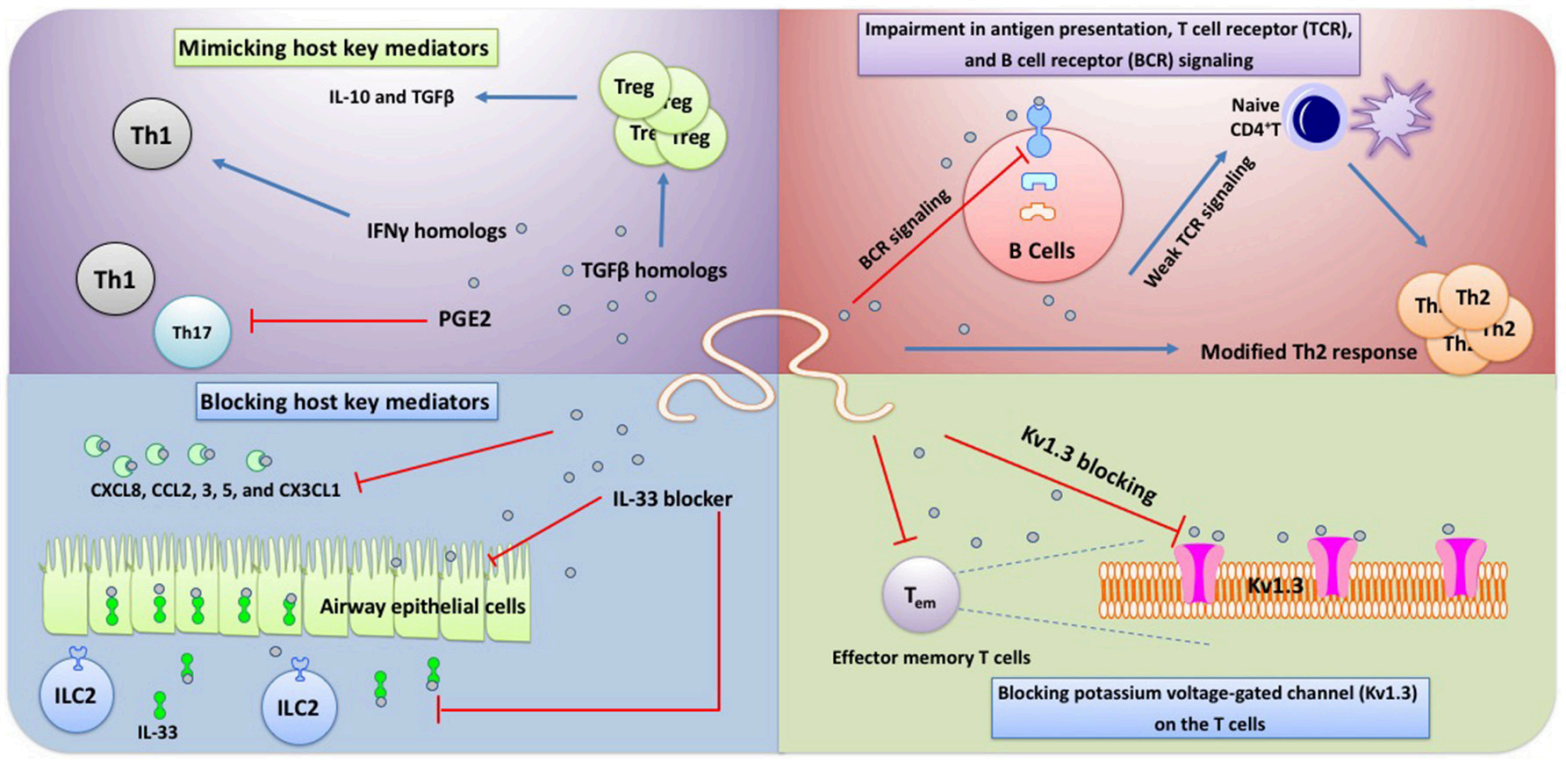
Some HDPs has been shown to target non-PRRs sensors including Kv1.3 and TCRs on T cells. Blocking Kv1.3 can significantly decrease Th1 cell activity and proliferation. Also, presenting HDPs on the MHCII leads to induction a weak TCR signaling in naïve CD4 T cells which corroborates Th2 differentiation. Of note, the main phenotype of Th2 response elicited by helminths and their products is “modified Th2” immunity in which IL-5, IL-13, eosinophlilia, and IgE are all downregulated, while IL-4, TGFβ, and IL-10 are increased. BCR signaling has also been shown to be impaired by some HDPs which prevent B cell activation. HDPs have recently been more considered as magic components which are able to mimic or block several host key mediators which play an important role in immunosuppression and Th2 amplification, respectively.

Generally, no precise pattern of intra- and extracellular sensors along with downstream signaling has been fully recognized by which HDPs exert immunomodulation. Indeed, helminths have shown to be able to exploit an array of highly complex strategies beyond the manipulation of canonical PRRs signaling to restore aberrant immune responses (136). In the following section, immunosuppressive functions of some HDPs through a PRR-independent manner will be discussed (Figure 2).

##### Impairment in antigen presentation, T cell receptor (TCR), and B cell receptor (BCR) signaling

Given the myriad biomolecules released by helminths, it is not surprising that some HDPs manipulate host immune cells via receptor-independent manner, such as enzymatic activity. For instance, *B. malayi* cystatins, such as Bm-CPI-1 (produced by the L2 and L3 stage of *B. malayi*) and Bm-CPI-2 are able to impede antigen presentation in APCs via interference with host cysteine protease function, reducing T cell priming (136, 137). In this way, Bm-CPI-2 was reported to inhibit human B cells to present tetanus toxoid associated peptide. Bm-CPI-2 was shown to possess two inhibitory sites enabling it to hinder host cysteine proteases and asparaginyl endopeptidase (AEP) (136–138). Likewise, *Onchocerca volvulus* was shown to diminish HLA-DR expression by release of onchocystatin (139, 140). Other worms, such as *Nippostrongylus brasiliensis* and *Litomosoides sigmodontis* produce cystatins enabling these worms to block B cell-associated endosomal proteases and T cell proliferation via IL-10 induction, respectively (140–143). However, the main mechanism through which helminth-derived cystatins affect host cells is unknown, but there is evidence suggesting the involvement of scavenger receptors and the transforming growth factor-β receptor (TGFβ R) pathways in particular by Avcystatin (*A. viteae*-derived recombinant cystatin) and calreticulin (a protein secreted by *H. polygyrus*), thereby polarizing Th2 responses (144, 145). In support of the critical role of helminth cystatins in promoting worm longevity, it has been suggested that presence of anti-cystatin circulating antibodies in mice can confine infection, suggesting that cystatins restrict the process of antigen loading and presentation by MHCII in APCs (141).

TCR signaling can also be affected by some HDPs in favor of shaping immune response toward immunoregulation (146–148). A well-known component of SEA known as omega-1 is the best example of HDPs which directs host immunity toward Th2 response through an intriguing mechanism. Mechanistically, omega-1 has been found to target cytoskeletal compartments to confine the formation of a stable DC-CD4^+^ T cell interplay, leading to suppression of TCR signaling strength and eventually Th2 cell differentiation. Since it has been documented that low dose antigens, which induce a weak TCR signaling, can prime Th2 response, the Th2-inducing property of omega-1 has also been ascribed to this hypothesis (149). Although omega-1 is able to inhibit TLR4-mediated DC stimulation independent of TLR-, MyD88- and TRIF, the main receptor targeted by omega-1 is still unknown, and it is thought that its enzymatic activity (T2 ribonuclease) likely contributes to such effects (86, 146). More investigations are required to identify whether other *S. mansoni* egg-derived antigens deviate immune responses toward Th2 by targeting TCR signaling (147). In support of helminth-mediated TCR signaling manipulation, Appleby et al. recently reported that in schistosome-endemic area of rural Zimbabwe, patients infected with *S. haematobium* had lower expression of CD3ζ (an integral part of TCR signaling) on peripheral blood mononuclear cells (PBMCs) than non-infected individuals, suggesting a possible TCR-targeted mechanism for hyporesponsiveness and immunosuppression (150).

In comparison to omega-1, the precise interruptive effects of ES-62 on TCR and BCR signaling have widely been determined (148). In fact, ES-62 by degradation of key components of downstream TCR and BCR signaling induces desensitization and in turn restrains cell proliferation and antibody production (151). Protein kinase C (PKC) and its different isoforms are one group of the major molecules that play a central role in orchestration of downstream cascade in TCR and BCR signaling for activation of transcription factors (152). It has been documented that ES-62 not only targets PKCs to be degraded/down-regulated in B and T cells but also up-regulates negative regulators of MAPKs signaling, such as RasGAP to suppress this pathway in Jurkat T cells (153). In addition, exposure of activated Th17 cells to LPS in the presence of ES-62 resulted in down-regulation of MyD88 and inhibition of LPS-mediated IL-17 production (154).

##### IgE signaling

Some HDPs, such as *Dirofilaria immitis*-derived antigen (DiAg) and *Clonorchis sinensis*-derived components called venom allergen-like proteins (CsVAL) are able to impair IgE-mediated degranulation followed by downstream signaling (155, 156). In this way, DiAg has been demonstrated to suppress type 1 diabetes in non-obese diabetic mice via Th1 inhibition and IL-10 induction (157). Interestingly, application of recombinant DiAg was associated with induction of nonspecific IgE by involving CD40 on B cells and saturation of FcεRI on mast cells, representing protective function against passive cutaneous anaphylaxis in rats (155). CsVAL has also displayed inhibitory activity on IgE-mediated degranulation of mast cells via targeting downstream pathways (156). Prevention of IgE receptor signaling has also been observed during exposure of human PBMCs to *S. mansoni* (158). It was revealed that the worm is able to secrete a component termed as schistosome-generated (SG) sCD23 which not only functions as a soluble decoy for the IgE receptor but also decreases the expression of this receptor (158). In addition, one of the well-characterized HDPs with interruptive effects on IgE signaling is ES-62. Collectively, these molecules might be considered as a potential therapeutic candidate in allergic disorders.

##### Mimicking and blocking host key mediators

TGFβ is one of the most powerful cytokines with immunoregulatory activity, which stimulates naïve T cells into regulatory T and B cells inducing immunobalance and tolerance in mucosal tissues, such as the intestine (159). This immunosuppressive cytokine and its receptor pathway have been reported to be mimicked and manipulated by helminths (159). Up to now, various components from nematodes like *A. caninum, B. malayi, H. polygyrus*, and trematodes, such as *F. hepatica* and the *Schistosoma* species have shown to produce TGFβ homologs (159). Bm-tgh-1 and Bm-tgh-2 from Brugia species and SmRK-1, 2, a TGF-β receptor homolog, from *S. mansoni* are well-known examples of HDPs with immunoregulatory activity due to mimicking TGFβ-induced mechanism (159). *H. polygyrus* is also able to stimulate naive T cells to express Treg-associated markers, such as Foxp3 amplifying immunoregulation via an unknown mechanism (160). Recently, Johnston et al. have characterized an important component from this worm known as *H. polygyrus* TGF-β mimic (Hp-TGM) which is structurally similar to a member of the complement control protein superfamily (161). Interestingly, Hp-TGM is functionally able to ligand mammalian TGFβR and mimic properties of TGFβ in induction of both mouse and human Foxp3^+^ Treg cells (161).

Furthermore, Sulaiman et al. have recently suggested that juvenile stages of *F. hepatica* release a TGF-like molecule known as FhTLM, engaging host TGF-β receptors and triggers Smad2/3 signaling in leukocytes (162). They showed that TGF-β RII is the main target of this component through which FhTLM increases the production of IL-10 and PD-L1 and the mannose receptor expression which eventually facilitate the early evasion of juvenile by suppression host immune activation (162).

Another class of host mediators which are masterfully mimicked by some helminths, such as *S. mansoni* (163), *Taenia taeniaeformis*, and *B. malayi* are prostaglandins (PGs) (164). Among various PGs, the immunoregulatory properties of PGE2 have widely been documented (165), and it is known that it can modulate DC function to polarize immune response from Th1 toward Th2 (166). Laan et al. have recently found that *T. suis* releases high levels of PGE2 in its excretory-secretory products which are likely responsible for inhibition of LPS-induced production of inflammatory cytokines from human DCs (25). By contrast, *T. muris* may expedite Th1 response by production of IFNγ homologs to engage IFNγ receptors leading to Th1 amplification and suppression of IL-4 mediated responses against the worm (167, 168). Of note, the immune response depends on the host genotype and the infection levels, for example, resistant genotypes display Th2 response while susceptible hosts represent Th1 response. Also, low infection levels of *T. muris* elicit a Th1 and high infection induces a Th2 response (168).

Another strategy whereby helminths attenuate inflammatory signals is production of bioactive blockers to occupy receptors and entrap mediators triggering inflammatory responses, such as chemokines (169). For instance, a well-known chemokine blocker is a chemokine-binding protein which is released by SEA called smCKBP (169). This component has been found to be able to bind several chemokine ligands including CXCL8, CCL2, 3, 5, and CX3CL1 resulting in suppression of both inflammatory signals and recruitment of inflammatory cells at the site of insult (169). Relevant to helminth-derived blocking compounds, a very recent study by Osbourn et al. recognized a potent IL-33 blocking component called *H. polygyrus* Alarmin Release Inhibitor (HpARI) (170). Mechanistically, HpARI binds to both nuclear DNA and mature IL-33, preventing its release and interaction with IL-33R on airway epithelial cells and ILC2. Further experiments in mice confirmed the suppression ability of HpARI on IL-33 and subsequent airway allergic responses, involving eosinophilia and IL-5 and−13 (170). These authors revealed a novel mechanism of suppressing Th2 response onset via inhibition of IL-33 released from necrotic epithelial cells which are insulted during helminth infection and exposure to airway allergens (170).

##### Blocking potassium voltage-gated channel (Kv1.3) on the T cells

It has recently been shown that some HDPs can control the function of T cells by blocking potassium voltage-gated channel (Kv1.3) (171). AcK1 and BmK1 are a large family of Stichodactyla helianthus toxin (ShK) composed of peptide structures secreted by *A. ceylanicum* and *B. malayi*, respectively. These compounds are able to block Kv1.3 channels on T cells by engaging the outer vestibule of the channel (171). Kv1.3 channels have been proved to widely be expressed and involved in activation of human T cells (172). Since there are an immense number of myelin-reactive T cells (autoreactive) resembling Kv1.3^high^ phenotype in multiple sclerosis (MS) forestalling these cells by targeting Kv1.3 channels would be of great interest (173, 174). In accordance with this, some clinical trial studies have been launched to evaluate the efficacy of AcK1 and BmK1 in attenuation of inflammation (NCT02446340; NCT02435342).

Taken together, these data show that a complex of diverse intracellular pathways is triggered by HDPs which ultimately converge host immunity toward hyporesponsiveness and immunological tolerance via manipulation of PRR-dependent and independent strategies. Up to now, we have briefly reviewed the intracellular interferences of HDPs as a foundation to provide novel insights into the possible mechanisms of extracellular vesicles (EVs) as an important component of HDPs. In the following section, we aim to discuss the recognized pathways utilized by EVs released during helminth infections to bypass host immunity with the aim of proposing other unknown mechanisms.

## Extracellular vesicles

### Biogenesis, content, and morphological properties

Various types of extracellular vesicles exist, but the structures that are most often referred to are called exosomes, which are 30–100 nm in diameter, or microvesicles, which measure about 100–1,000 nm in diameter (175). In terms of morphological properties and contents, exosomes are endocytic vesicles with a spherical shape surrounded by a bilamellar membrane. Their contents constitute a wide variety of biomolecules including glycans, proteins, lipids, and nucleic acids (176). Exosomes are originally derived from endosomes via budding from their wall, and then aggregated in a large body known as the multivesicular body (MVB). Exosomes are unleashed in extracellular environment upon exocytosis of MVB through fusion with the plasma membrane (176). Apart from exosomes, other distinct vesicles with similar function and properties can be detected in extracellular space including microvesicles (plasma membrane-derived microparticle) and apoptotic bodies, which can be distinguished from exosomes in terms of biogenesis, size, protein contents, density, and isolation technique (176). Also, the formation of microvesicles is simpler and happens through an outward budding of the plasma membrane (177). Generally, all of them are considered as EV and exosomes are the smallest EV with the lowest density (176). Apoptotic bodies are not the subjects of this review and beyond our discussion.

### Uptake in recipient cells

EVs have been acknowledged as important elements of cell-cell communication due to their role as vehicles for molecules and biological signals to be shuttled from one cell to another (178). Transfer of molecules, such as protein and miRNA can completely change the physiology of the receiving target cell and can in this way control both normal biological processes as well as distinct pathological processes (178).

EVs recognize and attach to their target cells through surface proteins, such as integrins, tetraspannins, proteoglycans, lectins, and immunoglobulins (179). The transfer of the EV content occurs as the EVs fuse with the target cells, which can be facilitated by various endocytic internalization pathways and the fusion can appear as rapidly as 15 min after exposure (180). Macropinocytosis is one such mechanism where EVs are captured and enclosed during the invagination of ruffled extensions from the plasma membrane (179). Another mechanism is phagocytosis, which is exploited by bacteria and viruses as well as DCs and macrophages and is facilitated by absorbing EVs by extended pseudopodia (180). Alternatively, EVs can be taken up by clathrin-mediated endocytosis, which involves the deformation of the target cell membrane by clathrin on the EV surface creating an inward bud that encloses the EVs, which can then pinch off and fuse with the endosome (181). Calveolin-dependent endocytosis is yet another mechanism for EV uptake where EVs oligomerize calveolin proteins on the membrane of the target cells facilitating the formation of intracellular calveolin-rich vesicles which then transports the EV contents to the target site (182). Finally, under specific conditions, EVs can fuse directly with the cell surface membrane (183).

Parasites use this remarkable ability of EVs to communicate with other parasite cells (parasite-parasite communication) (184) and host cells (host-parasite communication) (185). Strong evidence of the uptake of EVs by host cells has been obtained for a range of parasite species, including *Giardia duodenalis* (186), *Leishmania major* (187), *Plasmodium falciparum* (184), *Trichomonas vaginalis* (188), *Trypanosoma brucei* (189), *T. cruzi* (190), *Echinostoma caproni* (191), *F. hepatica* (28), *H. polygyrus* (30), *S. mansoni* (192), *A. suum, O. Dentatum*, and *T. suis* (Hansen et al., unpublished). Interestingly, Eichenberger et al. have recently employed organoids from murine colonic crypts to explore uptake of *T. muris-*derived EVs (193). These authors showed that when labeled EVs are delivered into the central lumen of organoids, they are internalized within 3 h and this process can be inhibited upon prevention of endocytosis (193). Furthermore, in this study transcriptomic and proteomic profile of *T. muris* EVs were characterized in which a number of miRNA with potential immunoregulatory effects on host immunity were identified (193). Upon fusion, specifically loaded compounds and biological signals in the EVs are transferred, which then can exert its function in the target cell. These events are responsible for the EV-related host immune manipulation (30, 194, 195), pathogenesis (196, 197) and parasite development (198), that have been reported for many parasitological diseases as described previously.

## EVs for the host immune system

### EV-mediated delivery of message by helminths

A number of parasites have shown to release EVs and they have received great interest due to their remarkable immunomodulatory properties including suppression of IL-6, TNFα, and IL-17A in mice (191, 199, 200). Various potential mechanisms have been suggested through which these vesicles might affect host cells (201). In fact, they have unique biological properties mediating cell-cell interaction both via direct contact and/or delivering biomaterial contents affecting intracellular crosstalk (202). However, the specific intracellular mechanisms that are targeted remain unknown or only partially characterized.

In recent years, helminth-associated EVs have shown to be essential mediators that contain bioactive cargo that contribute to establishing a permissive infection (30, 202). Apart from helminths, other microbes including fungi, bacteria, and protozoa are able to produce EVs and mediate active interaction with their host immunity to achieve a suitable niche (203). The release of such a protected package can undoubtedly optimize the integrity of contents, which in turn improve their efficacy in genetic exchange and secure delivery to modulate host immunity (203). Indeed, several studies have shown that powerful biomolecules are present in the helminth-derived EVs, which can offer potential and druggable candidates against inflammatory diseases (204). In addition, omics-based approaches, such as proteomics and transcriptomics have provided a plethora of valuable data facilitating recognition of major components mediating host immunosuppression. Such explorations provide novel insight into previously unknown strategies by which worms send messages to host cells to manipulate the host immune system and may therefore also pave the way to new ways to control infections (204–206).

Given interesting results achieved from EVs investigation, a surge of interest has been paid to focus on their contents. In the following, the most important biomolecules represented by helminth EVs along with their potential implications are discussed.

### miRNAs as an important player for intracellular communication

One important component of EVs is miRNA, which is an evolutionary conserved class of short non-coding RNA, of 19–24 nucleotides in length, which plays an essential role in post-transcriptional gene regulation and is involved in the fine-tuning of a range of cellular processes. miRNA has been discovered in all types of human body fluids (207) and in various different species (208), including parasitic organisms (209, 210).

miRNA has been shown to take part in the regulation of any kind of biological process, here among embryonic development (211), cell differentiation and apoptosis (212–214) but also various immunological processes (215). In fact, miRNA seems to be involved in basically all aspects of immunological events of both innate and adaptive character. That being differential and activation of macrophages (216), dendritic cells (217), granulocytes (218), NK cells (219), and other innate immune cells (220) as well as development and function of B cells (221, 222) and T cells (223, 224).

Recent investigations suggest that helminth EVs also contain various RNA molecules including miRNA which may participate to the manipulating signaling pathways which orchestrating inflammatory responses (193). This mechanism may enable worms to directly interfere with host gene translation and thereby immunity to facilitate parasite survive (29, 30, 225). In support of this, an *in vitro* study showed that *E. multilocularis* produces a type of miRNA (emu-miR-71) which is able to suppress NO production and inflammation-associated miRNAs in LPS/IFN-treated murine macrophage RAW264.7 (29). By contrast, the effects of *S. japonicum*-derived exosomes on the same cell line of macrophages showed an increase of iNOS and TNF-α, supporting M1-type macrophage development (226). The steadily growing body of literature is supporting the critical role of exosome-associated miRNA as an important player involved in suppression or stimulation of target cells (30). One of this miRNA-mediated mechanism which has been recently suggested by Buck et al. is genetically blocking or targeting host mRNA for degradation, causing a severe defect in host immune response to confine worm expulsion. Molecular digestion showed that this miRNA is able to target 3′ untranslated region of the mRNA encoding Th2-associated cytokines, such as IL-33 which in turn prevents the ILC2 expansion and eliciting a strong Th2 response (30). Thus, it seems that helminths possess EVs with bioactive compounds, such as miRNAs that are able to effectively neutralize host immunity and polarize immune responses toward the way supporting their survival.

Gu et al. also suggest that some helminth miRNAs only might target host genes and that their release is a selective process as the profile of released miRNAs in the excretory-secretory products differed from that of adult extracts of *Haemonchus contortus* (227).

In addition to miRNA, other RNA molecules have been identified within helminth EVs, such as mRNA and tsRNA (193, 228) but their potential function and role in host-parasite interaction still need to be elucidated.

### Proteins and lipids in EVs

In addition to nucleic acid, EVs do also contain a diverse collection of molecules including lipids and proteins (229, 230). The protein composition has been characterized in excretory-secretory products from several parasite species using proteomic approaches based on mass spectrometry and has shown that it contains a suite of immunomodulatory proteins with immune suppressive effects e.g., on type 1 and type 2 effector molecules (30, 231–233).

Proteins identified in EVs comprise several peptidases, proteases, cytoskeletal proteins, nuclear proteins, calcium-binding proteins as well as stress-related proteins (28, 234, 235). Importantly, Coakley et al. demonstrated that *H. polygyrus* secretes EVs containing Argonaute protein, which is a central protein in the RNA-induced silencing complex (RISC) and is a powerful mechanism of gene silencing (229, 236). Furthermore, EVs are expected to be an important part of the secretome and it has been shown that the secreotomes of many helminths contain a variety of highly-abundant leaderless proteins, which is homolog to damage-associated molecular pattern molecules (DAMPs) in the host (28, 197). These DAMPs can, just like the host's DAMPs, modulate the host innate cells, such as stimulation of cytokine secretion in macrophages and lymphocytes. This could indicate that helminths have evolved mechanisms that are similar to their hosts in order to avoid elimination by the immune response of the hosts (182, 237).

Interestingly, besides parasite proteins, EVs have also been shown to contain host-derived proteins. Marcilla et al. identified 36 different host proteins in vesicles from the trematode *E. caproni*, which mainly corresponded to histones, partial sequences of mucins, metabolic enzymes, and immunoglobulins (28). Their study suggests that EVs might be an important part of the host-parasite communication as host proteins were identified in the parasite EVs and as the vesicles were taken up by host cells. These findings are interesting, however further studies are needed to confirm the role of the vesicles for the establishment of the infection (28). Very recently a similar study has been conducted by Eichenberger et al. in which proteomic profile of *T. muris*-derived EVs also identified a number of proteins of importance in host-parasite interaction. Of interest, among the common most proteins were predicted not to have a signal peptide supporting the idea that EVs constitute an important mechanism by which molecules are transferred to the host (193).

Surprisingly, several studies have shown that a significant proportion of the EV proteins are lacking an N-terminal presequence, also referred to as transit peptide. The transit peptide is required for protein transport across relevant membranes and indicates a protein transport through a classical sorting pathway via ER/Golgi pathway (238). Since a significant proportion of the parasite proteins are lacking the transit peptide, this suggests that EVs may be an important mechanism for transport of proteins host cells (28, 185, 239).

Lipids are a major component of EVs and play an important role in the function, stability, rigidity, and uptake of EVs, as they are directly exposed to the environment. Most of the lipidomic studies have been performed in cancer cells and these studies have indicated that EVs may have a function as lipid carriers where they carry bioactive lipids to recipient cells. In the context of the tumor microenvironment, this transport may have an effect on the enrichment of tumor progression and immunosuppressive lipids, such as prostaglandins that enhance tumor growth (240, 241). Even though lipids are central to EV trafficking, only a few studies have yet explored the lipid content of EVs in parasites and their specific role remain to be elucidated (242, 243).

## EVs as novel vaccines

Even though helminth parasites infect over 25% of the world's population and are highly prevalent in livestock worldwide, these infections are one of the most “neglected” tropical diseases with no effective vaccines available for humans and only a few for animals (244, 245). The use of EVs as vaccines is an area of growing interest due to their immunomodulation role and EVs ability to generate specific antibodies (30, 191, 202).

Using EVs purified from *Echinostoma caproni*, Trelis et al. found that subcutaneous injection could reduce symptom severity and mortality of subsequent experimental infection in mice (191). Likewise, EV vaccination in mice generates specific antibodies and was sufficient to confer protective immunity against *H. polygyrus* challenge (202). *T. muris*-derived EVs has also recently been found to confer protection against subsequent infection (246). Several proteins were identified in the *T. muris* EVs and some proposed to be potential candidates for vaccine (246).

However, further studies are needed to further explore the potential of parasite-derived EVs as novel vaccine candidates (206). In another approach, DCs have been stimulated or infected with protozoan parasites following isolation of EVs, which then have been tested as vaccines. In this way, DC-derived EVs have shown to confer protective immunity against *Toxoplasma gondii* (247), *Leishmania major* (248).

Sotillo et al. observed that 31% of the identified proteins in EVs secreted from *S. mansoni* are homolog to previous describes vaccine candidates, where several of these proteins are common throughout the life cycle of the parasite. This indicates that a vaccine based on EVs could target different life stages of the parasite and may be effective against *S. mansoni* infection (249). However, EVs are biological complex and virtually nothing is known about their specific mechanisms and how they engage with the immune system. However, it was recently demonstrated that EVs from *H. polygyrus* are able to suppress both the activation of macrophages and target the Il-33 pathway, which is known to be essential for worm expulsion (202). Taken together, all these observations indicate that EVs hold a great potential for future vaccine development, and thereby pave the way for novel treatment options against parasite infection (206).

## EVs as novel generation of biomarkers: possibilities and potentials for early diagnosis of parasitic infections

EVs have shown to contain a rich source of molecules and as these are protected from the environment by a membrane they are stable over time (250). In addition, as EVs are distributed in various body fluids and organs, they have received great interest for biomarker-based diagnosis (250). Indeed, the potential of EVs as a biomarker has widely been investigated in the field of cancers and the contents of exosomes derived from ovarian cancer patients have shown to differ from that of healthy individuals (250). Interestingly, some cancer types are associated with specific miRNA signatures in the exosomes and may, therefore, serve as a novel way of diagnosis and monitoring progression of treatment (251, 252). In infectious diseases, EVs isolated from serum samples have also shown potential in diagnostics (253). However, the physical location of the pathogen may affect the applicability of this method or at least the way samples should be collected. Buck et al. found that miRNA of *Litomosoides sigmodontis*, which is located in the pleural cavity, could be identified in the host serum whereas no miRNA of *H. polygyrus*, which resides in the gut lumen could be detected (30).

With the advent of high throughput assays, which now also includes EV array (254), a profound understanding is being established regarding host-parasite interplay and the main pathway by which EVs target host immune cells. Proteomics analysis on the HDPs concentrated in EVs has provided invaluable data to identify and purify the central candidate components for biomarkers, diagnostic tools, and vaccine development (255, 256). Likewise, HDPs merely have shown an acceptable potential to be considered as an approach to early infection diagnosis (257). For instance, cathepsin B1 from *Opisthorchis viverrini* has shown a potential candidate for further consideration as a biomarker in sera (258).

**Figure 3:**
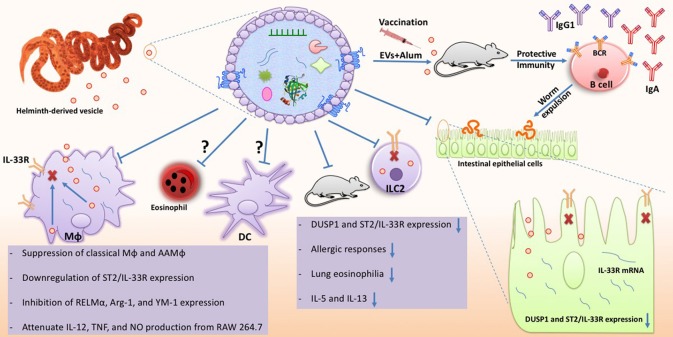
Schematic illustration of known effects imposed by helminth-derived EVs during interaction with host immunity. As illustrated here, EVs can affect different host cells including immune cells and intestinal epithelial cells (IECs). Also, they can potentially be used to develop new vaccines against helminths. EVs deliver cargo containing various biomolecules to host cells which can interfere with host cell gene transcription. EVs are internalized by macrophages and IECs via unknown mechanism and target IL-33R and DUSP1 expression reducing signal transmission and leads to inhibition of helminth expulsion. EVs have also shown great potential in priming host immunity along with Alum as a vaccine complex against helminth infections. Effective suppression of both classical Mϕ and AAMϕ have also been reported, implying EVs are well-equipped with a wide range of active components. In addition to *in vitro* studies, immunomodulatory functions of EVs have also been monitored in *in vivo* model in which allergic responses, associated cytokines, ILC2, and eosinophilia are down-regulated in Alternaria-exposed mice.

Altogether, the release of EVs might be a reciprocal beneficial interaction for both host and helminths. Eliciting an effective immune response against EVs is an obvious reason for the presence of highly immunogenic components which may offer possible biomarker candidate. On the other hand, helminths constantly explore a way to defeat host immunity and set up a permissive infection which production of EVs containing various types of material is might be a strong strategy (Figure 3).

## Perspectives

Until now, many different and highly complex mechanisms have been suggested for immunosuppressive functions of HDPs. But, given the advances in recognition of EVs and their importance as an effective tool in manipulation of host immune cells, it makes sense to suppose the possible involvement of EVs in most HDPs-mediated immunomodulation.

Main intracellular pathways manipulated by HDPs were reviewed, but limited information has been provided regarding interaction between helminth-derived EVs and immune cells. Although several investigations have recently reported the potential intracellular functions of EVs, they are only partially characterized. Given the different mechanisms by which EVs are taken up by recipient cells (discussed in EV section), future investigations should focus on the intracellular mechanisms and pathways responsible for immunosuppression.

We believe that most mechanistic pathways which have been reported for HDPs can be tracked for EVs and their contents. Most biomolecules form contents of EVs require to reach into the recipient cells to show their functions. The intracellular and PRR-targeting mechanisms of HDPs which were discussed in this review provide a framework to explore the possible interaction between EVs contents and intracellular components. In this regard, study on the PRRs-EV interplay would be of great interest to address some of the intracellular effects mediated by EVs. Release of EVs cargo in host immune cells can be associated with various outcomes, so determining the potential interaction between EVs cargo and PRRs downstream signaling would be valuable. For instance, Kojima et al. (259) showed that mesenteric lymph is able to exacerbate inflammatory responses via activation of TLR4 on macrophages without uptake through this receptor. They also showed that TLR4 signaling pathway is essential for exosome-induced macrophage activation (259).

EVs have recently been considered as a promising option to tune immune responses, as well as reprogram aberrant reactions to innocuous antigens. With respect to this, unraveling the main components of helminth-derived EVs, which are responsible for host immunomodulation may offer personalized therapeutic approaches in translational medicine.

Also, from a diagnostic point of view, EVs released by helminths may pave the way for developing a rapid diagnostic test that also can detect early parasitic infection. In addition, recognition and targeting main players in the biogenesis of helminth-derived EVs will be associated with novel insights into control of parasitic infections. Obviously, suppression of EVs synthesis containing a wide array of bioactive components will neutralize one of the central mechanism of helminths to overcome host immunity. Interestingly, interfering with EVs biogenesis with exploiting selective inhibitors has provided promising results in the field of cancer therapy (260), but the applicability of this approach to combat helminth infections remains elusive. However, several investigations have been conducted to assess the potential of protozoan EVs for vaccine development against toxoplasmosis and leishmaniasis (248, 261). In these studies, exosomes derived from DCs exposed to parasite antigens have shown to be able to protect against infection in experimental models. Collectively, it seems that the importance of EVs and their relevance in establishment of a chronic infection are less-known and eagerly awaited to precisely be unraveled in the near future.

## Author contributions

AZ was the major contributor in conception, design, illustrations, and writing of the manuscript. EH and SA wrote the EV section. PN and AW edited the manuscript and provided critical comments, suggestions, and insightful revisions. This work was supervised by PN.

### Conflict of interest statement

The authors declare that the research was conducted in the absence of any commercial or financial relationships that could be construed as a potential conflict of interest.
